# Study on the Conventional Performance and Microscopic Properties of PPA/SBS-Modified Bio-Mixed Asphalt

**DOI:** 10.3390/ma15124101

**Published:** 2022-06-09

**Authors:** Guiyong Liu, Wei Zhang, Xiaolong Yang, Zhikang Ning

**Affiliations:** 1 School of Civil Engineering and Architecture, Guangxi University, Nanning 530004, China; 2010302050@st.gxu.edu.cn (G.L.); xiaolongyang@gxu.edu.cn (X.Y.); 3127083804@st.gxu.edu.cn (Z.N.); 2 Gansu Road & Bridge Construction Group Co., Ltd., Lanzhou 730099, China

**Keywords:** bio-asphalt, PPA, SBS, conventional performance, micromorphological

## Abstract

To promote the construction of environmentally friendly, sustainable pavements and solve the impact of the scarcity of asphalt resources on highway development, bio-mixed asphalt (BMA) modified by SBS and polyphosphoric acid (PPA) was prepared, and the influence of the ratio of bio-asphalt (BA) replacing petroleum asphalt on different PPA/SBS blending schemes was explored through conventional property tests. According to each PPA/SBS blending scheme, the optimal replacement ratio of bio-asphalt was optimized, and the microstructure and distribution morphology of different PPA/SBS-modified BMA were evaluated. Conventional property test results show that with the same PPA/SBS content, the replacement ratio of bio-asphalt has a significant impact on the conventional performance of composite-modified asphalt, but the appropriate replacement ratio of bio-asphalt can improve the storage stability and conventional performance of composite-modified asphalt; in micromorphological analysis, it was found that the number of bee-like structures on the surface of the modified BMA decreased significantly, which indicated that the molecular heterogeneity of various components in the asphalt was reduced. In addition, bio-asphalt changed the particle morphology and improved the dispersity of SBS in asphalt. The composite-modified BMA had a lower SBS content, but its conventional performance was still excellent—so it has significant application prospects in road engineering.

## 1. Introduction

Asphalt has become the primary choice of cementitious materials for pavement construction by the virtue of its good skid resistance, driving comfort and easy maintenance. However, with the development of highway construction in various countries, the demand for asphalt binder is increasing rapidly, but the scarcity of petroleum resources has led to difficulties in meeting the demands of road construction for asphalt production. Therefore, it is urgent to seek a new material to deal with the impact of insufficient petroleum asphalt capacity caused by the lack of crude oil resources, and the appearance of bio-asphalt brings a new idea for solving the above problems. In addition, as an environment-friendly and sustainable material, the application of bio-asphalt in road engineering provides an effective method for the treatment of bio-waste.

The raw materials of bio-asphalt come from a wide range of sources, including waste by-products from agriculture and animal husbandry—such as excrement, straw, millet bran and waste wood [1,2]—and through a series of pyrolysis, separation and purification, the sustainable bio-waste above can be prepared to make bio-asphalt [3]. Although bio-asphalt has made it possible to construct low-carbon, sustainable and environmentally friendly asphalt pavement, many researchers have found that the performance of bio-asphalt or bio-mixed asphalt is poor and finds it difficult to meet the performance requirements of pavements under heavy traffic loads. Chen et al. explored the diffusion performance of bio-asphalt based on molecular dynamics and found that adhesion performance decreased significantly with the increase of bio-asphalt content [1]; Yang et al. also came to similar conclusions [4]. Williams et al. used waste wood and straw to prepare bio-asphalt and partially replaced petroleum asphalt to explore the mechanical properties of bio-mixed asphalt. They found that bio-asphalt had good compatibility with petroleum asphalt, but the bio-mixed asphalt had poor high-temperature performance [5]. Gao, Mohamed and Mills-Beale et al., based on rheological tests, found that the rutting resistance of asphalt was significantly reduced under the action of bio-asphalt [6,7,8]. In summary, due to the large number of highly active hydroxyl compounds in the bio-asphalt, it is easily soluble in the light components of the asphalt, resulting in a significant decrease in the high-temperature performance of the bio-mixed asphalt [9]. In addition, some researchers point out that wood-based bio-asphalt has a certain negative impact on the low-temperature cracking resistance of petroleum asphalt [10]. Therefore, the instinctive limitations of bio-asphalt and the performance degradation of petroleum asphalt caused by the addition of bio-asphalt prevent the application of the bio-asphalt in pavement construction.

At present, styrene-butadiene-styrene block copolymer (SBS) is widely used in the preparation of high-performance modified asphalt [11,12]. However, the high content of hydroxyls with strong polarity in bio-asphalt improves the compatibility of SBS and asphalt, which can significantly elevate the modification efficiency of SBS modifiers [13]. Therefore, the combination of SBS and bio-asphalt has good application prospects. However, some researchers have pointed out that the modification of SBS on bio- or bio-mixed asphalt is a physical effect and does not form stable chemical bonds [14,15], and the intermolecular polarity decreases with increases in temperature. The above reasons result in a significant segregation phenomenon that appears in SBS-modified asphalt. In contrast, polyphosphoric acid (PPA), as a chemical modifier, can not only react with asphalt to form stable chemical bonds, but also has the advantages of high modification efficiency, good storage stability, low price, etc.—the most important being that it can significantly improve the high-temperature performance of asphalt [16,17,18]. In addition, the natural molecular polarity of bio-asphalt can significantly improve the ionization degree of PPA—thus further improving the modification efficiency of PPA [19]. In conclusion, due to the poor mechanical properties of bio-asphalt, its application in road engineering is limited to some extent. SBS and PPA can not only significantly improve the high-temperature performance of bio-mixed asphalt, but also the interactions between bio-asphalt, PPA and SBS, with a positive effect on the improvement of each other’s performance. At the same time, the application of renewable and environmentally friendly bio-asphalt in road engineering solves the problem of agricultural waste management and addresses the sustainable and clean development of road engineering, improves the efficiency of resource utilization and achieves economic, social and environmental benefits.

This study was conducted to investigate the influence of the ratio of bio-asphalt replacing petroleum asphalt on the conventional performance of different PPA/SBS blending schemes. According to each PPA/SBS blending scheme, the optimal replacement ratio of bio-asphalt was optimized, and the microstructure of different PPA/SBS composite-modified BMA was evaluated through atomic force microscopy and fluorescent microscopy—thus promoting the application of bio-asphalt in high-performance pavement construction.

## 2. Materials and Methods

### 2.1. Materials

The Shell 90# paving asphalt (AS) was obtained from the Shell Petroleum Asphalt Factory, Tianjin, China. The physical properties of the asphalt were as follows: softening point: 46.4 °C (ASTM D36); penetration: 86.6 dmm (25 °C, ASTM D5); ductility: 69.3 cm (10 °C, ASTM D 113); Rotational viscosity: 0.44 Pa·s (135 °C, ASTM D 4402). The selected PPA industrial grade polyphosphate, with a P_2_O_5_ content of 83.31%, and its physicochemical properties are shown in Table 1. Bio-asphalt (BA) is a renewable plant asphalt made of starch and straw from the Tianjin Haoxiang Chemical Co., LTD; its basic properties are provided by the manufacturer (shown in Table 2). SBS is a linear polymer with 30% styrene and 70% butadiene.

### 2.2. Preparation of the Modified Asphalt

In the preparation of SBS-modified asphalt, a 4% SBS content is wildly used; so, this study selected 4% SBS-modified asphalt as the control group. In addition, the preliminary experimental study showed that the content of PPA does not benefit from exceeding 1.5%; so, 0.5%, 1% and 1.5% PPA were selected to partially replace the SBS in order to prepare composite-modified asphalt.

The preparation process of the composite-modified BMA was as follows:

1. **Step 1 bio-mixed asphalt preparation:** biological and petroleum asphalt were heated to a fluid state, then mixed in a specific ratio and stirred at 1000 rpm for 20 min;
2. **Step 2 SBS modification:** the bio-mixed asphalt was raised to 170 ± 5 °C with continuous stirring, then SBS was added and sheared at 5000 rpm for 40 min, and the stabilizer (sulfur flour, 0.2% of binder by weight) was added in the last 10 min;
3. **Step 3 PPA modification:** PPA was added and sheared at 5000 rpm for 30 min (SBS control group shear for 30 min without PPA addition);
4. **Step 4 Swelling development:** the prepared composite-modified BMA was placed in a vacuum drying oven at 170 °C for 1 h.

**Figure 1:**
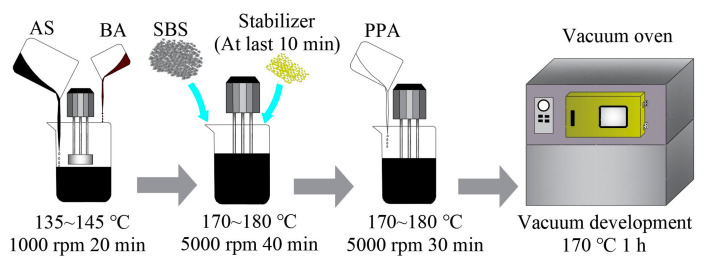
Preparation process of BA/PPA/SBS-modified asphalt.

**Figure 2:**
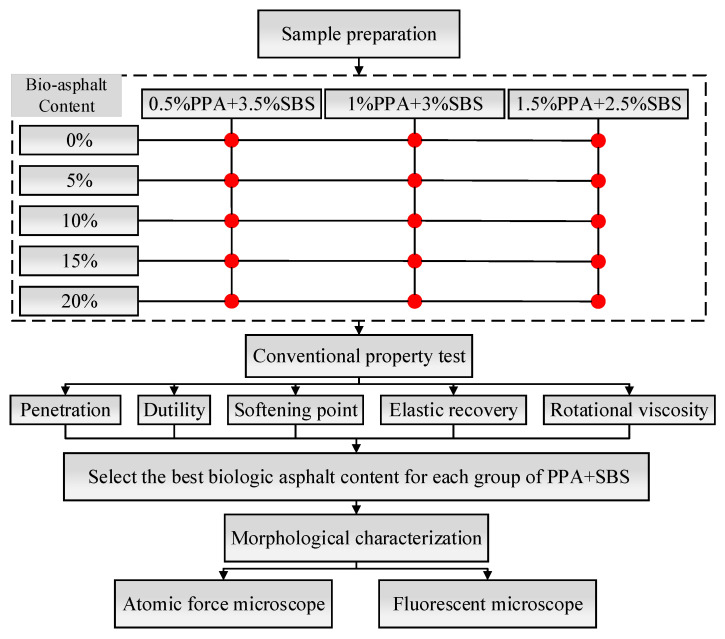
Flowchart of the research.

The specific preparation process is shown in Figure 1 below. A flowchart of this research is presented in Figure 2.

### 2.3. Test Methods

#### 2.3.1. Conventional Property Test

Conventional property tests of the composite-modified BMA, including penetration (25 °C, 100 g, 5 s), ductility (5 °C), softening point, elastic recovery, rotational viscosity and storage stability tests, were conducted according to ASTM D 5, D 113, D 36, D 6084, D 4402, and D 5976, respectively.

#### 2.3.2. Atomic Force Microscope (AFM)

An AFM from BRUKER Dimension FastScan (Bruker, Billerica, MA, USA) was used to explore the micromorphology of different asphalt samples by using the tapping mode at a scanning area of 15 × 15 μm. The specimens were prepared by the hot pouring method [20,21]. Specifically, the composite-modified BMA was heated to a completely flowing state and then poured into a metal container with an inner diameter of 16 mm and a height of 3.5 mm. Then, the specimens were heated for 10 min in an oven at 120 °C to smoothen the surface. Finally, the specimens were cooled to room temperature (about 25 °C) in a dustproof cover.

#### 2.3.3. Fluorescent Microscope (FM)

The swelling behavior of SBS in asphalt is an important factor affecting the mechanical performance of SBS-modified asphalt. Therefore, this study investigated the phase structure of PPA/SBS composite-modified BMA based on falling fluorescence microscopy (CAIKON, DFM-66 C). In order to obtain the real SBS particle phase structure, FM samples were prepared according to the research conclusions of Kang et al. [22,23], and the composite-modified asphalt was heated to flow state (170 ± 5 °C), and then dropped into a metal ring with a diameter of 150 mm and a height of 3 mm based on quartz glass. Finally, the specimens were heated for 10 min to ensure a smooth surface, and finally cooled to room temperature in a dustproof cover.

## 3. Results and Discussion

### 3.1. Analysis of the Conventional Property Test

**Figure 3:**
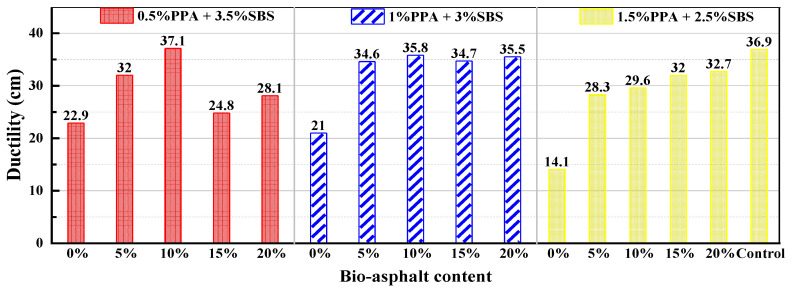
Test results for ductility.

The penetration test results for the composite-modified BMA with different mixing schemes are shown in Figure 3, in which the bio-asphalt content is the percentage of bio-asphalt in the total binder. According to the figure, the ductility of PPA/SBS-modified asphalt without BA is significantly lower than that of SBS-modified asphalt. However, the ductility of asphalt with different PPA/SBS mixing schemes has a different variation trend after the BA addition. For the 0.5% PPA + 3.5% SBS group, its ductility increased first and then decreased with increases in BA content, and it reached its maximum when the BA content was 10%. The maximum ductility of the 1% PPA + 3% SBS group was the same as when the BA content was 10%, but its ductility did not change significantly with increases in BA content. As for the 1.5% PPA + 2.5% SBS group, the variation trend in ductility increased with the rise in BA content, and reached its maximum when the BA content was 20%.

**Figure 4:**
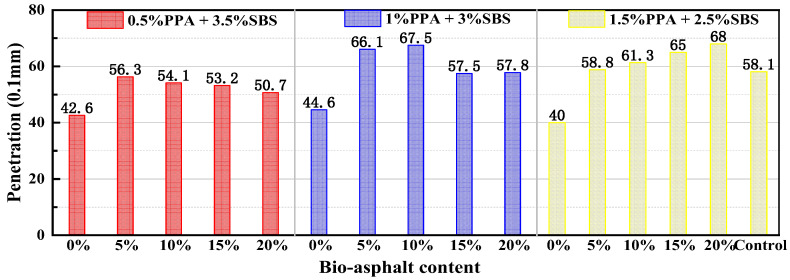
Test results for penetration.

The penetration test results are shown in Figure 4. In general, the penetration of modified asphalt in each group increased with decreases in SBS content. For the 0.5% PPA + 3.5% SBS, it had the largest penetration when the bio-asphalt content was 5%, while the 1% PPA + 3% SBS and the 1.5% PPA + 3% SBS had the largest penetration in BA with 10% and 20% bio-asphalt content. However, the penetration of the composite-modified BMA showed no significant change compared to that of the control SBS-modified asphalt.

**Figure 5:**
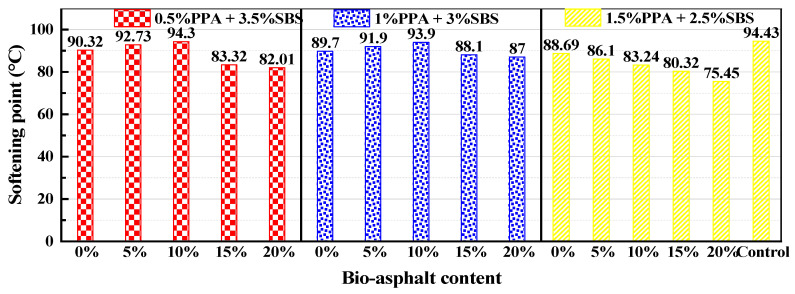
Test results for the softening point.

The softening point test results of the modified asphalt in each group with different BA content are shown in Figure 5. The composite-modified asphalt with three kinds of PPA/SBS blending schemes showed excellent high-temperature performance under the optimum BA replacement ratio. Among them, the softening point of the 0.5% PPA + 3.5% SBS and the 1% PPA + 3% SBS groups reached its maximum when the BA content was 10%, which is equivalent to the control group. However, the softening point of the 1.5% PPA + 2.5% SBS group decreased with increases in BA content.

**Figure 6:**
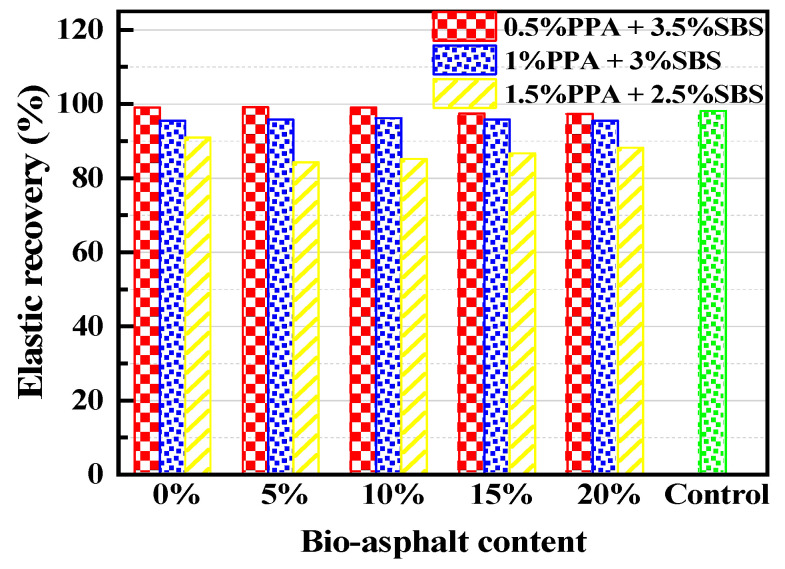
Test results for elastic recovery.

The elastic recovery test results are shown in Figure 6. It can be seen from the figure that the elastic recovery of asphalt in each group decreased with reductions in SBS content, but when the SBS content decreased by 12.5%, 25% and 37.5%, the elastic recovery of the asphalt in the three groups decreased by only 0.1%, 2% and 9% compared with the control group—indicating that PPA had a certain positive effect on the elastic recovery of the asphalt. Liu and Zhou et al. obtained the same results [16,24]. In addition, the improvement of the BA replacement ratio had a small impact on the group with the 3.5% and 3% SBS content, but in general, BA had a small impact on the elastic recovery of asphalt, with a maximum decrease of less than 5%.

**Figure 7:**
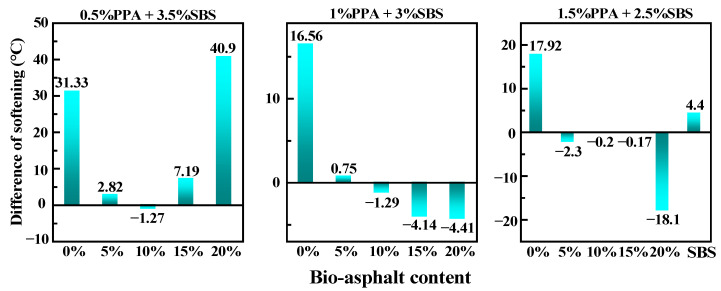
Test results for segregation.

A storage stability test of the PPA/SBS-modified asphalt with different BA content was conducted and the results are shown in Figure 7. The segregation of composite-modified asphalt was significantly reduced after adding BA. It is worth noting that the segregation intensified when the PPA content was 0.5% and the BA content was more than 10%. When the PPA content was 1%, the negative segregation appeared (the softening point of the bottom was larger than that of the top), and it intensified with increases in BA content. When the PPA content was 1.5% and the BA content was 20%, the negative segregation was the most significant. The reason for this may be that the alcohols’ functional groups in the BA have natural molecular polarity, which improves the swelling performance of SBS particles, and because the condensation and esterification of PPA increase the density of SBS micelles—thus reducing the density difference between the SBS phase and asphalt phase. In addition, the boundary thickness of SBS particles and whether chemical bonds are formed have a great influence on the storage stability of SBS-modified asphalt [25], while BA not only improves the boundary thickness of SBS particles, but also produces chemical cross-linking between SBS and asphalt due to its esterified reaction with PPA. Therefore, under the optimal BA content, its storage stability is significantly improved, while when the PPA and BA content is too high, the density of the SBS and BA micelles will be significantly increased—resulting in significant negative segregation.

**Figure 8:**
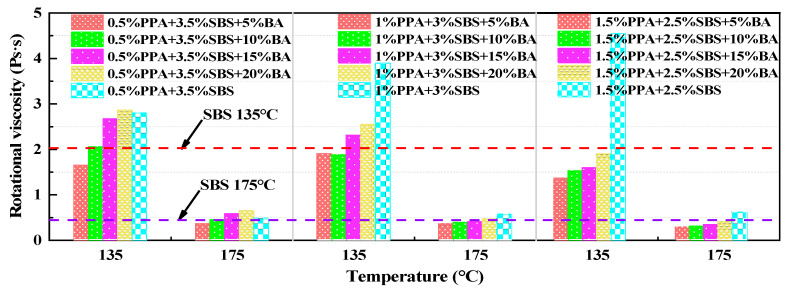
Test results for rotational viscosity.

The rotational viscosity test results are shown in Figure 8. According to the figure, decreasing SBS content can significantly reduce asphalt viscosity. In addition, by comparing the viscosity of modified asphalt in the same group with different dosages of BA, it was found that increasing the dosages of BA will improve the viscosity of asphalt to a certain extent, but the viscosity of all composite-modified BMA was less than 3 Pa·s at 135 °C, and the viscosity of the composite-modified asphalt with appropriate dosages of BA was lower than that of the control group.

In conclusion, the SBS content of the three groups of PPA/SBS-modified asphalt decreased by 12.5%, 25% and 37.5% compared with the control group, respectively. However, the performance of the composite-modified asphalt did not significantly decrease after the addition of BA, and even improved under some replacement ratios. Therefore, the PPA/SBS composite-modified BMA has significant economic significance.

Taking the performance requirements of the JTG F40 [26] for polymer-modified asphalt and maximizing the replacement ratio of BA to petroleum asphalt as the optimization principles, the conventional performance of the three groups of PPA/SBS-modified asphalt under different BA content was comprehensively analyzed, and the following three composite-modified BMA blending schemes were finally optimized (as shown in Table 3). The performance of the three groups of composite-modified asphalt met the I-D or I-C performance requirements in the JTG F40.

Then, a micromorphological analysis was conducted for three kinds of composite-modified BMA and SBS-modified asphalt (control group).

### 3.2. Microstructure Analysis of the Composite-Modified Asphalt

**Figure 9:**
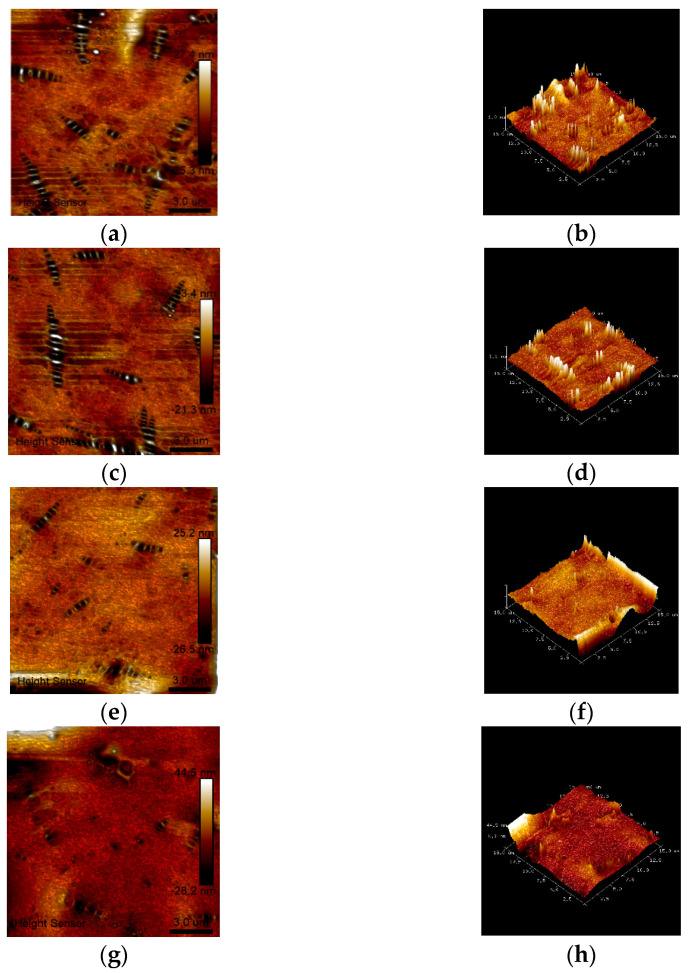
Topography maps of different samples: (**a**) 2D topography (A1); (**b**) 3D topography (A1); (**c**) 2D topography (A2); (**d**) 3D topography (A2); (**e**) 2D topography (A3); (**f**) 3D topography (A3); (**g**) 2D topography (A4); (**h**) 3D topography (A4).

The 2D and 3D surface topography of each asphalt sample is shown in Figure 9. The bee-like structure in the figure is the convex surface structure generated by asphaltene micelles or other substances (such as SBS particles), with different physical and chemical properties from the light component that cannot be dissolved and dispersed [27,28,29]. By comparing the 2D morphologies of various asphalt samples, it was found that the number of bee-like structures on the surface of asphalt obviously decreased with increases in the ratio of BA + PPA to SBS, and the surface morphologies became smoother in the 3D morphologies. Previous studies have shown that under the action of PPA, asphaltene increases and resin decreases in petroleum asphalt [30,31], which often leads to an increase in the molecular weight of asphalt micelles and a significant increase in the bee-like structure on the surface topography. However, the BA used in this study is derived from straw, with a high content of polar alcohol functional groups that are more likely to react with PPA to generate esterification [19]. This not only reduces the influence of PPA on each component of the petroleum asphalt, but also improves the molecular weight of the SBS micelles after absorbing BA under the action of PPA esterification and condensation and reduces the intermolecular heterogeneity. In addition, high molecular heterogeneity may cause the material to be prone to fracture [32], so the addition of BA can improve the cracking resistance of composite-modified asphalt—this corresponds to the high m-value of composite-modified BMA (which represents the stress dissipation capacity of asphalt at low temperatures) in subsequent studies. However, too much PPA and BA will lead to the excessive molecular weight of the SBS micelles, so there is a negative segregation phenomenon in the segregation test. In conclusion, BA has a significant effect on reducing the molecular heterogeneity among the components of asphalt, which may be the reason for the better storage stability of composite-modified BMA.

### 3.3. Phase Structure Analysis of the Composite-Modified Asphalt

**Figure 10:**
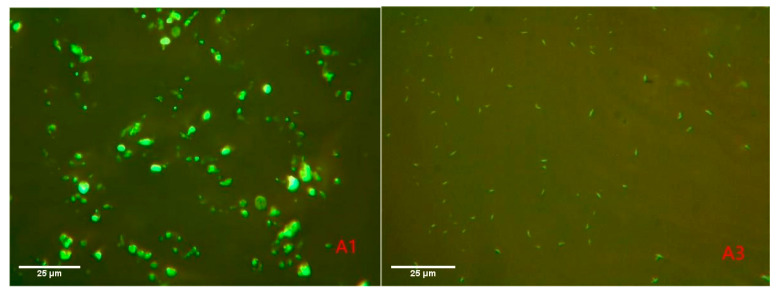
Fluorescence images of different modified asphalts.

The dispersion and phase structure of the SBS modifier in asphalt were observed by FM. The fluorescence images of the control and A3 group after amplification 400 times are shown in Figure 10. The fluorescence phase in the figure was emitted by SBS particles after absorbing ultraviolet light. Therefore, it can be seen from Figure that SBS particles in A1 group were larger and showed a clumpy structure. Under the condition of the same amplification ratio, the shapes of the SBS particles in the A3 group with BA were completely different from those in A1, with an elongated feathery structure and good dispersion. This phenomenon also confirmed that the phase difference between the SBS particles and asphalt was reduced after SBS fully absorbed the bio-asphalt, and so the composite-modified BMA was relatively smooth in its surface topography. In addition, smaller SBS particles have a larger surface area, which improves the action efficiency of SBS modifiers. Therefore, the slight reduction in SBS content did not result significantly decrease the conventional performance of the composite-modified BMA. Furthermore, the storage stability of SBS is guaranteed under the action of PPA, but the action mechanism of PPA needs further study to be determined.

**Figure 11:**
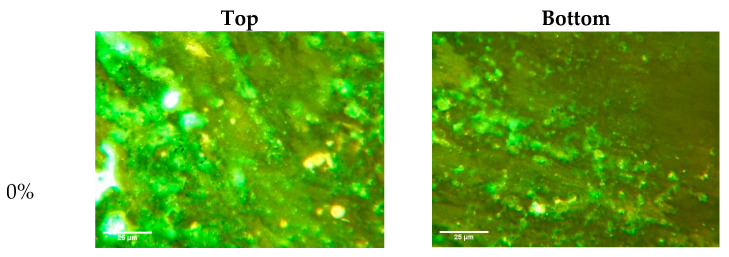

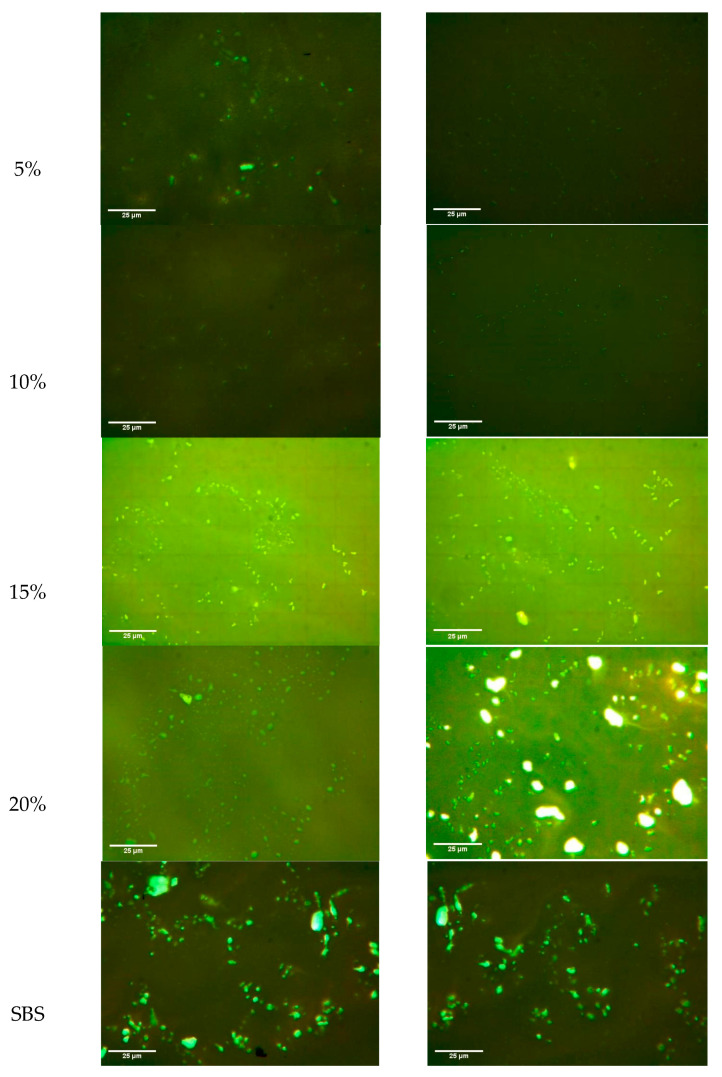
Fluorescence images of different asphalts after the segregation test.

Under the condition of 1% PPA + 3% SBS modifier content, the segregation phenomenon in composite-modified BMA with different BA replacement ratios was explored. The fluorescence images of the different modified asphalts are shown in Figure 11. When the BA replacement ratio was 0%, a large number of clusters and impurities appeared in the FM diagram—which may be due to PPA reducing the content of light components of petroleum asphalts, leading to a significant decrease in the dispersion and solubility of the SBS and stabilizer [25]. After the addition of BA, the SBS particles showed good dispersion, but when the dosage of BA and PPA was not appropriate, the SBS particles still appeared to be agglomerated; for example: when the BA replacement ratio was 20%, there was obvious agglomeration and a high fluorescence intensity in the bottom. In general, there is a corresponding relationship between the content of SBS and BA with PPA. When the content of BA is insufficient, the positive segregation of SBS particles may occur (the softening point of the upper tube is large), while when the content of BA is too high, negative segregation may occur (the softening point of the lower tube is large).

## 4. Conclusions

In order to promote the construction of environment-friendly and sustainable bio-asphalt pavement, this research studied the conventional performance and microscopic morphology characteristics of PPA/SBS composite-modified asphalt with different bio-petroleum asphalt mixture ratios, and drew the following conclusions:

(1) Through conventional property tests, it was found that the replacement ratio of bio-asphalt had a significant impact on the conventional performance of composite-modified asphalt, but under the optimal content of bio-asphalt, the high and low-temperature performance of the composite-modified asphalt was similar to that of the SBS-modified asphalt. In addition, BA and PPA can improve the storage stability of SBS-modified asphalt.
(2) Compared with the SBS-modified asphalt, the surface topography of the composite-modified asphalt with bio-asphalt had a higher flatness, indicating that bio-asphalt had a certain positive effect on reducing the molecular heterogeneity among the components of the asphalt.
(3) Bio-asphalt significantly improved the dispersity of the SBS in asphalt and changed the phase structure of the SBS in asphalt from clumpy to feathered. Although bio-asphalt and PPA improved the storage stability of the SBS-modified asphalt, the SBS particles would accumulate to the bottom when the dosage of the bio-asphalt and PPA was too high.
(4) In this study, the SBS content of PPA/SBS-modified bio-mixed asphalt was significantly lower than that of single-doped SBS-modified asphalt, but its conventional performance was not significantly lower than that of SBS-modified asphalt. In addition, the application of bio-asphalt in road engineering provides an effective solution for the disposal of waste biomass. Therefore, as a kind of renewable and sustainable material, bio-asphalt has good application prospects in road engineering.
(5) The reaction mechanism of each component had prominent significance for the performance optimization and engineering application of the composite-modified asphalt. Therefore, the investigation of the chemical reaction mechanisms of each component of the composite-modified asphalt should be the focus of future research.

## Figures and Tables

**Table 1:** Physicochemical properties of polyphosphoric acid.

Characteristics	Unit	Value
Chroma (once)	-	10
H_3_PO_4_	%	115.5
P_2_O_5_	%	83.31
Chloride	%	0.00005
Fe	%	0.0004
As	%	0.006

**Table 2:** Physical properties of the bio-asphalt.

Characteristics	Unit	Value
Appearance	-	Dark brown
Relative density (25 °C)	g/cm^3^	1.24
Kinematic viscosity (100 °C)	mm^2^/s	240
Pour point	°C	24
Flash point	°C	265
Volatility (163 °C, 3 h)	%	0.05
Moisture	%	0.02

**Table 3:** The proportion of Bio-asphalt/PPA/SBS-modified asphalt.

Proportion Scheme	Namely
4% SBS + AS (100)	A1
0.5% PPA + 3.5% SBS + AS:BA (90:10)	A2
1% PPA + 3% SBS + AS:BA (90:10)	A3
1.5% PPA + 2.5% SBS + AS:BA (85:15)	A4

## References

[1] Chen W., Chen S., Zheng C. (2021). Analysis of micromechanical properties of algae bio-based bio-asphalt-mineral interface based on molecular simulation technology. Constr. Build. Mater..

[2] Shao L., Wang H., Zhang R., Zheng W., Hossiney N., Wu C. (2021). Analysis of the chemical properties and high-temperature rheological properties of MDI modified bio-asphalt. Constr. Build. Mater..

[3] Yang X., Mills-Beale J., You Z. (2017). Chemical characterization and oxidative aging of bio-asphalt and its compatibility with petroleum asphalt. J. Clean. Prod..

[4] Yang X., You Z., Mills-Beale J. (2015). Asphalt binders blended with a high percentage of biobinders: Aging mechanism using FTIR and rheology. J. Mater. Civ. Eng..

[5] Williams R.C., Satrio J., Rover M., Brown R.C., Teng S. Utilization of fractionated bio-oil in asphalt. Proceedings of the Transportation Research Board Annual Meeting.

[6] Junfeng G., Hainian W., Zhanping Y., Mohd M.H., Yong L., Muhammad I. (2018). Rheological behavior and sensitivity of wood-derived bio-oil modified asphalt binders. Appl. Sci..

[7] Mohamed R., Williams R. (2010). Temperature and shear susceptibility of a nonpetroleum binder as a pavement material. Transp. Res. Rec. J. Transp. Res. Board.

[8] Mills-Beale J., You Z., Fini E., Zada B., Lee C.H., Yap Y.K. (2014). Aging influence on rheology properties of petroleum-based asphalt modified with biobinder. J. Mater. Civ. Eng..

[9] Oyebanji J.A., Okekunle P.O., Lasode O.A., Oyedepo S.O. (2017). Chemical composition of bio-oils produced by fast pyrolysis of two energy biomass. Biofuels.

[10] Xu Y., You Z.P., Dai Q.L. (2013). Performance evaluation of asphalt binder modified by bio-oil generated from waste wood resources. Int. J. Pavement Res. Technol..

[11] Wen G., Zhang Y., Zhang Y., Sun K., Fan Y. (2002). Rheological characterization of storage-stable SBS-modified asphalts. Polym. Test..

[12] Tayfur S., Ozen H., Aksoy A. (2007). Investigation of rutting performance of asphalt mixtures containing polymer modifiers. Constr. Build. Mater..

[13] Dong Z., Yang C., Luan H., Zhou T., Wang P. (2019). Chemical characteristics of bio-asphalt and its rheological properties after CR/SBS composite modification. Constr. Build. Mater..

[14] Yvonne M.B., Müller A.J., Rodriguez Y. (2003). Use of rheological compatibility criteria to study SBS modified asphalts. J. Appl. Polym. Sci..

[15] Navarro F.J., Partal P., Martínez-Boza F., Gallegos C., Bordado J.C.M., Diogo A.C. (2006). Rheology and microstructure of MDI–PEG reactive prepolymer-modified bitumen. Mech. Time-Depend. Mater..

[16] Liu H.Y., Chang R., Cao X.J., Hao P.W. (2017). Road performance of polyphosphoric acid composite modified asphalt mixture. J. Build. Mater..

[17] Li C., Cui S.C., Wang L., Bai X.F. (2020). Rheological properties of polyphosphoric acid/SBS composite modified asphalt at high temperature. Mater. Rev..

[18] Baldino N., Gabriele D., Lupi F.R., Oliviero Rossi C., Caputo P., Falvo T. (2013). Rheological effects on bitumen of polyphos-phoric acid (PPA) addition. Constr. Build. Mater..

[19] Masson J.F. (2008). Brief review of the chemistry of polyphosphoric acid (PPA) and bitumen. Energy Fuels.

[20] Wang M., Liu L. (2017). Investigation of microscale aging behavior of asphalt binders using atomic force microscopy. Constr. Build. Mater..

[21] Xing C., Liu L., Wang M. (2019). A new preparation method and imaging parameters of asphalt binder samples for atomic force microscopy. Constr. Build. Mater..

[22] Kou C.J., Wu X., Kang A.H., Liu Y. (2020). Quantitative analysis of fluorescent microscopic image of SBS modified asphalt. Chin. Sci. Technol. Pap..

[23] Kang A.H., Zhang Y.C., Wu H., Sun L.J. (2012). Preparation method of modified asphalt samples for fluorescence microscopic ob-servation. J. Sichuan Univ..

[24] Liu S., Zhou S., Peng A. (2020). Evaluation of polyphosphoric acid on the performance of polymer modified asphalt binders. J. Appl. Polym. Sci..

[25] Zhang F., Hu C. (2013). The research for SBS and SBR compound modified asphalts with polyphosphoric acid and sulfur. Constr. Build. Mater..

[26] (2017). Technical Specifications for Construction of Highway Asphalt Pavement.

[27] Loeber L., Sutton O., Morel J., Valleton J.-M., Muller G. (1996). New direct observations of asphalts and asphalt binders by scanning electron microscopy and atomic force microscopy. J. Microsc..

[28] Yang J., Gong M.H., Troy P., Wei J.M., Wang X.T. (2015). Study on microstructure of asphalt based on atomic force microscopy. Acta Pet. Sin..

[29] Yang J., Wang X.D., Gong M.H., Chen J. (2015). Characteristic analysis of atomic force microscope microscopic image of asphalt. Acta Pet. Sin..

[30] Wang L., Cui S.C., Ren M.D. (2019). Evaluation of microstructure properties of polyphosphoric acid compound SBS modified asphalt. Mater. Rev..

[31] Yan K., Zhang H., Xu H. (2013). Effect of polyphosphoric acid on physical properties, chemical composition and morphology of bitumen. Constr. Build. Mater..

[32] Ma W., Huang T., Guo S., Yang C., Ding Y., Hu C. (2019). Atomic force microscope study of the aging/rejuvenating effect on asphalt morphology and adhesion performance. Constr. Build. Mater..

